# Is geriatric depression scale a valid instrument to screen depression in Chinese community-dwelling elderly?

**DOI:** 10.1186/s12877-021-02266-y

**Published:** 2021-05-13

**Authors:** Feifei Huang, Huijun Wang, Zhihong Wang, Jiguo Zhang, Wenwen Du, Xiaofang Jia, Liusen Wang, Bing Zhang

**Affiliations:** National Institute for Nutrition and Health, Chinese Center for Disease Control and Prevention, No. 27, Nanwei Road, Xicheng District, Beijing, China

**Keywords:** Geriatric, Depression, Mild cognitive impairment, Epidemiology, Psychometrics

## Abstract

**Background:**

The geriatric depression scale (GDS) is used widely as a screening instrument for depression worldwide. The present study aims to examine the reliability and validity of the GDS with 30 items (GDS-30) in Chinese cognitively normal elderly, and to preliminarily investigate the appropriateness of the GDS-30 among screened mild cognitive impairment (MCI) elderly and among the large-scale community-dwelling Chinese elderly.

**Methods:**

A total of 12,610 Chinese elderly completed GDS-30 in the project of Community-based Cohort Study on Nervous System Diseases. Of these, 5503 individuals with the ability to perform basic daily living activities were randomly sampled to further complete the Montreal Cognitive Assessment to screen for MCI. The cutoff value of screened depression was 11, and the cutoff values of MCI were education-dependent. Internal consistency was used to evaluate the reliability. Exploratory factor analysis (EFA) was used to determine the factor structure. Confirmatory factor analysis (CFA) was conducted to assess the construct validity in the elderly screened normal cognition, screened MCI, and the whole population, respectively.

**Results:**

The Kuder-Richardson coefficient (KR20) was 0.834, 0.821 and 0.840 for the cognitively normal elderly, screened MCI and the whole population, respectively. EFA showed that GDS-30 can be either a four-factor model (named positive mood, dysphoria, worry, and social withdrawal-cognitive impairment) or a two-factor model (named depression and positive mood). The latter was easier to interpret. CFA showed that the two-factor model fitted well in the elderly with normal cognition, with screened MCI, and the whole sample. The factors loaded from 0.900 to 0.588, 0.882 to 0.529, and 0.888 to 0.556 in these three populations respectively.

**Conclusions:**

The GDS-30 has good reliability and validity and can be appropriately applied to screen depression in the large-scale community-dwelling Chinese elderly regardless of the presence of mild cognitive impairment.

## Background

Depression is a common mental disorder that cannot be ignored. More than 264 million people suffer from depression globally in 2017 [1]. From 1990 to 2007, the number of all-age years lived with disabilities (YLDs) attributed to depression increased by 33.4 %, from 31.0 to 35.8, becoming the third leading cause of all-age YLDs in 2007. Between 2007 and 2017, the YLDs further increased by 14.3 %, becoming the third and fifth leading cause of all-age YLDs for females and males respectively in 2017 [1]. Depression is one of the important causes of suicide and a leading cause of disability worldwide. The lack of treatment is due to the barrier to effectively and correctly diagnose, which is complicated, time-consuming and must be completed by a professional psychiatrist. A convenient self-report instrument is needed to screen depression in the large-scale population.

Beck Depression Inventory (BDI) [2], Hamilton Depression Scale (HAM-D) [3], Center for Epidemiological Studies Depression Scale (CES-D) [4], Patient Health Questionnaire-9 (PHQ-9) [5], Cornell Scale for Depression in Dementia (CSDD ) [6] and Zung Self-rating Depression Scale (SDS) [7] were all widely used self-report screening instruments for depression. However, these scales were not designed for elderly specifically, some problems had happened when applied to elderly. Firstly, the response format was too complicated for the elderly to answer. For example, some scales had multiple choices for each question, asking the participants to choose the one closest to the actual situation. Other scales asked participants to estimate the frequency that each description happened. Secondly, somatic symptoms, which might be caused by other physical disorders, were not specific to depression. Scales containing questions about somatic symptoms, such as decreased appetite or sleeping disorders, would overestimate the prevalence of depressive symptoms. Considering these existing problems, Brink and Yesavage developed the Geriatric Depression Scale (GDS), a 30-item screening questionnaire specifically designed for the elderly [8, 9]. None of the 30 items was somatic, thus avoiding the confusion of somatic symptoms with physical disturbances that were common in the elderly [10]. The dichotomous response of yes or no format was easier for the elderly to select an answer. Some Shorter versions of the GDS had been developed as well, such as the GDS with 15 items (GDS-15) [11], with 10 items (GDS-10) [12], with 5 items (GDS-5) [13], with 4 items (GDS-4) [12] and with 1 item (GDS-1) [12].

The GDS with 30 items (GDS-30), validated and used around the world in many languages [14–17], was a reliable and valid screening instrument, although the factor structure varied across different language versions [18]. A systematic review reported that the sensitivity was 0.753 and specificity was 0.770 of the pooled GDS-30 studies [19]. The earliest studies on GDS-30 in China mainly focused on the elderly in Hong Kong. In 1994, Chiu et al. firstly examined the reliability, validity and factor structure of GDS-30 for Chinese elderly in Hong Kong and found that the reliability and validity were satisfactory [20]. And then, Chan et al.’s study revealed that the reliability and validity of GDS-30 were exceptional, and the sensitivity (70.6 %) and specificity (70.1 %) were acceptable for Hong Kong elderly [21]. Chau et al. also found that the GDS-30 had acceptable reliability with 0.88 for Cronbach’s alpha and excellent convergent and divergent validity in a Cantonese-speaking Hong Kong Chinese group of stroke patients [22]. Also, the GDS-30 also had good reliability and validity in the community sample of elderly in Chinese-American immigrants [23], Chinese rural community-dwelling elderly in Hunan province [24], Chinese urban community-dwelling elderly in Beijing [25], Chinese elderly in Sichuan province [26], and Chinese elderly in Hunan, Beijing and Shandong provinces [27].

However, some limitations exist in the current studies among the Chinese elderly. Firstly, the sample size was small, with 113 [20], 461 [21], 253 [22], 50 [23], 412 [24], 397 [25], 383 [26], 1553 [27] elderly respectively, which was not representative of the general population in China. Secondly, the GDS-30 produced structures with one to seven and nine factors respectively when used among distinct populations with different languages [9, 20, 24–34]. The most standard procedure of validity verification procedure was to conduct the exploratory factor analysis (EFA) and confirmatory factor analysis (CFA) successively. Most of the studies conducted either EFA [24, 25, 28, 30–35] or CFA [22, 29], which were incomplete and with relatively poor methodological quality. Finally, geriatric depression often occurs in the context of cognitive impairment and dementia, thus it was difficult to screen depression symptoms by GDS-30 among elderly with cognitive impairment or dementia because of the symptom overlap. There was no consensus that whether GDS-30 could be used among elderly with cognitive impairment[33, 36, 37]. To our knowledge, most studies that validated the GDS-30 among Chinese elderly failed to use samples of cognitively intact or describe the cognitive characteristics of the sample except Huang’s study [30]. This study aimed (1) to examine the reliability and validity of the geriatric depression scale with 30 items in Chinese elderly with screened normal cognition, and (2) to preliminarily investigate the appropriateness of the geriatric depression scale with 30 items among screened mild cognitive impairment (MCI) individuals and large-scale community-dwelling Chinese elderly, respectively.

## Methods

### Study sample

The participants were from the project of Community-based Cohort Study on Nervous System Diseases (CCSNSD) subordinated to the Cohort Study on Nervous System Diseases which was a National Key Research and Development Program of China, Precision Medicine Project. The project was undertaken by the National Institute for Nutrition and Health of the Chinese Center for Disease Control and Prevention, and cooperated by the Center for Disease Control and Prevention of Zhejiang, Shaanxi, Hunan provinces, Hebei Medical University and Xuan Wu Hospital of the Capital Medical University. Considering the discrepancy between the north and the south of China, the project drew a sample with a multistage, random cluster sampling method from Hebei, Zhejiang, Shaanxi and Hunan provinces. Each province consisted of two cities and two counties. One urban neighborhood and one suburban village were chosen randomly from each city, and one county neighborhood and one rural village were chosen randomly from each county, respectively. Patients diagnosed with epilepsy, Parkinson’s disease, or Alzheimer’s disease were excluded before the project.

The project’s baseline survey was conducted between 2018 and 2019 through face-to-face interviews by trained investigators, except the self-reported GDS-30. A total of 12,610 individuals aged 55 years old and above completely finished the GDS-30. We randomly selected 5588 of them to assess further whether they were MCI or not. Of these, 85 participants were excluded for their inability to perform basic daily living activities involving eating, dressing, bathing, toileting, grooming, transferring bed or chair, walking across a room, and urinary or fecal continence. The remaining 5503 elderly conducted the Montreal Cognitive Assessment (MoCA), of which 1902 were screened as MCI.

### Measurements

The GDS used in this study consisted of 30 items with a dichotomous response of yes or no. Twenty items indicated depressive symptoms with the answer of ‘yes’, and 10 items indicated depressive symptom with the answer of ‘no’. The score of each item was 1 for the answer representing depressive symptom and was 0 for the answer not representing depressive symptom. The total score was obtained by summing across all the items, with a higher score indicating greater depressive symptoms. The developer of GDS-30 reported that a score of 11 as the cutoff value gave a sensitivity of 0.84 and a specificity of 0.95, and a score of 14 gave a sensitivity of 0.80 and a specificity of 1.00. We adopted 11, recommended by the developer and employed in most studies, as the cutoff value for screening depressive symptoms [9, 38].

We used MoCA, developed by Ziad S Nasreddine in 2005, to screen MCI in this study [39]. It was a 30-point test covering eight cognitive domains, with a higher score indicating better cognitive function. The short-term delayed memory recall task scored 5, which asked the participants to learn five nouns read by the investigators and then recall them in approximately five minutes later. The visuospatial abilities scored 4, with a clock-drawing task (3 points) and a three-dimensional cube copy (1 point). Executive functions scored 4, with an alternate connection task (1 point), a phonemic fluency task (1 point), and a two-item verbal abstraction task (2 points). Attention, concentration, and working memory scored 6, which were evaluated by a sustained attention task (tapping the desk when listening to the target number; 1 point), a serial subtraction task (minus 7 for five consecutive times, 3 points), and repeating digits forward and backward (1 point each). Language abilities scored 5, asking the participants to name three low-familiarity animals (lion, giraffe, camel; 3 points), and to repeat two syntactically complex sentences (2 points). Finally, orientation to time and place scored 6. The cutoff value for screening as MCI was not agreed upon worldwide. Adherence to the developer’s and some researchers’ suggestions, the score will plus 1 if the year under education was not more than 12 [39, 40]. As recommended by a population-dwelling study in China, we regarded ≤ 13 for illiterate individuals, ≤ 19 for those with primary education, and ≤ 24 for those with at least junior high education as the cutoff values when screening for MCI [40].

### Statistical analysis

The internal consistency reliability of the GDS-30, performed by SPSS 26.0, was examined by Kuder-Richardson coefficient (KR20), and the reasonable acceptability criterion was ≥ 0.70 [41]. We calculated the KR20 in the elderly with normal cognition, with screened MCI, and the whole sample, respectively.

The construct validity was examined by EFA and CFA successively. We sorted the participants with screened normal cognition (*n* = 3601) in ascending order according to their unique personal identification numbers. Participants in the odd lines were grouped into sample 1 (*n* = 1801) and those in the even lines were grouped into sample 2 (*n* = 1800). Firstly, EFA was conducted in sample 1 using SPSS 26.0. We performed Kaiser-Meyer-Olkin (KMO) measure of sampling adequacy and Bartlett’s test of sphericity to test the feasibility of factor analysis, and then performed the principal component analysis (PCA) with orthogonal varimax rotation for the GDS-30 to extract factors with eigenvalues ≥ 1. Items with a factor loading of > 0.35 were considered to contribute to the factor. Secondly, CFA was conducted in sample 2 using Mplus 8.3. Given that the 30 items had a binary responses, the maximum likelihood method was not appropriate. We used the robust weighted least squares with mean and variance adjustment (WLSMV) estimator [42]. Models with comparative fit index (CFI) > 0.90, Tucker-Lewis index (TLI) > 0.90 and root mean square error of approximation (RMSEA) ≤ 0.08 were regarded as acceptable [43]. Because standardized root mean square residual (SRMR) was susceptible to sample size, especially when the outcome was binary response, we did not use SRMR to assess the goodness of fit of our models [44].

After confirming the factor structures of GDS-30 in the elderly with screened normal cognition, the CFA was re-conducted in the screened MCI individuals (*n* = 1902) and the whole sample (*n* = 12,610) respectively to test the appropriateness.

The mean ± sd for continuous variables with normal distribution, median (p25, p75) for continuous variables with abnormal distribution, and percentages for categorical variables were presented to describe the study population using SAS 9.4. Independent-samples T-test was used for continuous variables with normal distribution, Chi-square test was used for categorical variables, and Mann-Whiteney U test was used for continuous variables with abnormal distribution. All statistical tests were two-tailed and employed a significance level at *P* < 0.05.

## Results

### Descriptive statistics

Table 1 presented the descriptive statistics of our sample. The whole sample consisted of 12,610 individuals with a mean age of 67.3 years and a median total score of GDS-30 for 4. Of the sample who conducted the MoCA (*n* = 5503), 1902 (34.6 %) were defined as MCI, and the remaining 3601 individuals were defined as normal cognition. The depressive symptoms rate, which was 9.3 % for participants with normal cognition and 10.2 % for participants with screened MCI, was statistically equal (*P* = 0.1441). Regarding the whole sample, 9.5 % of the participants were screened as having depressive symptoms, of which 93.6 % were mild and 6.4 % were moderate to severe. The median scores of GDS-30 were 4, 4 and 5 for the whole sample, participants without or with screened MCI, respectively.

**Table 1:** Sample demographics

	Whole sample	Participants with normal cognition	Participants with screened MCI	*P* ^†^
Sample size (n, %)	12,610 (100.0)	3601 (100.0)	1902 (100.0)	-
Age (y, mean ± SD)	67.3 ± 7.6	66.6 ± 7.1	68.6 ± 7.7	< 0.0001^‡^
Gender (n, %)				
Male	5441 (43.2)	1612 (44.8)	826 (43.4)	0.3422^§^
Female	7169 (56.8)	1989 (55.2)	1076 (56.6)	
GDS-30 score (P50, (P25, P75))	4 (2, 8)	4 (2, 9)	5 (2, 9)	< 0.0001^¶^
Degree of depressive symptoms (n, %)				
No	11,412 (90.5)	3267 (90.7)	1708 (89.8)	0.1441^§^
Mild	1122 (8.9)	318 (8.8)	178 (9.4)	
Moderate to severe	76 (0.6)	16 (0.5)	16 (0.8)	
Education-adjusted MoCA score (P50, (P25, P75))	24 (19, 28)	27 (23, 30)	18 (14, 21)	< 0.0001^¶^

^†^Statistical test between participants with or without cognitive impairment

^‡^Independent-samples t’-test

^§^Chi-square test

^¶^Mann-Whiteney U test

The heterogeneity between sample 1 and sample 2 was shown in Table 2. Age, gender, GDS-30 score and degree of depressive symptoms showed no statistical difference. It is reasonable to conduct the EFA in sample 1 and CFA in sample 2.

**Table 2:** Heterogeneity comparison between sample 1 and sample 2

	Sample 1	Sample 2	*P*
Sample size (n, %)	1801 (100.0)	1800 (100.0)	-
Age (y, mean ± sd)	66.6 ± 7.1	66.7 ± 7.1	0.6764^†^
Gender (n, %)			
Male	791 (43.9)	821 (45.6)	0.3075^‡^
Female	1010 (56.1)	979 (54.4)	
GDS-30 score (P50, (P25, P75))	4 (2, 9)	4 (2, 9)	0.7011^§^
Depressive symptoms (n, %)			
No	1626 (90.3)	1641 (91.2)	0.8870^‡^
Yes	175 (9.7)	159 (8.8)	

^†^Independent-samples t-test

^‡^Chi-square test

^§^Mann-Whiteney U test

### Internal consistency reliability

For the elderly with normal cognition, the KR20 of GDS-30 was 0.834. When removing each item of the GDS-30 from the analysis separately to test the robustness, KR20 remained high, from 0.822 to 0.838.

For the participants with screened MCI, the KR20 of GDS-30 was 0.821. When removing each item of the GDS-30 separately, KR20 remained high, from 0.809 to 0.830.

In the whole sample, the KR20 of GDS-30 was 0.840. When each item of the GDS-30 was deleted from the analysis, KR20 remained high, from 0.830 to 0.846.

### Exploratory factor analysis

PCA with the maximum variance orthogonal rotation was performed in sample 1. The KMO value for the GDS-30 was 0.923, and Chi-Square of the Bartlett’s sphericity test was 18030.092 (*P* < 0.001). Four factors were extracted according to the criterion of eigenvalue ≥ 1.0 and they accounted cumulatively for 47.356 % of the total variance. After rotation, the four factors explained 16.686 %, 11.662 %, 10.969 and 8.039 % of the total variance, respectively.

The item loadings were shown in Table 3. Factor 1, representing positive mood, consisted of 10 items (item 21, 7, 9, 27, 19, 5, 29, 15, 30 and 1) which were all positively descriptive sentences, with factor loadings ranging from 0.789 to 0.427. Factor 2 (item 3, 4, 6, 8, 10, 2, 11 and 13) was defined as dysphoria, with factor loadings from 0.678 to 0.398. Factor 3 (item 25, 24, 22, 18, 16 and 17) was interpreted as worry, with factor loadings from 0.746 to 0.514. The remaining 6 items (item 20, 14, 23, 28, 26 and 12) pertained to the factor 4, social withdrawal-cognitive impairment, with factor loadings from 0.703 to 0.469. Each of the five items (item 11, 12, 13, 17 and 18) showed cross-loadings on two factors, but was retained and assigned to the factor on which they loaded most highly. The secondary loading was shown in parentheses.

**Table 3:** Factor loadings for EFA after rotation in sample 1

Item	Factor 1	Factor 2	Factor 3	Factor 4
21. Full of energy	0.789			
7. In good spirits most of the time	0.773			
9. Feel happy most of the time	0.764			
27. Enjoy getting up in the morning	0.762			
19. Find life very exciting	0.721			
5. Hopeful about the future	0.705			
29. Easy to make decisions	0.685			
15. Wonderful to be alive now	0.684			
30. Mind as clear as it used to be	0.662			
1. Basically satisfied with life	0.427			
3. Feel life is empty		0.678		
4. Often get bored		0.674		
6. Bothered by thoughts		0.651		
8. Afraid something bad will happen		0.599		
10. Often feel helpless		0.549		
2. Drop many activities and interests		0.534		
11. Often get restless and fidgety		0.512	(0.415)	
13. Frequently worry about the future		0.398	(0.394)	
25. Frequently feel like crying			0.746	
24. Frequently get upset over little things			0.713	
22. Situation is hopeless			0.610	
18. Worry a lot about the past		(0.379)	0.566	
16. Often feel downhearted and blue			0.559	
17. Feel pretty worthless the way		(0.397)	0.514	
20. Hard to get started on new projects				0.703
14. Have more problems with memory than most				0.609
23. Most people are better off				0.579
28. Prefer to avoid social gatherings				0.512
26. Have trouble concentrating				0.492
12. Prefer to stay at home, rather than going out and doing new things		(0.375)		0.469
Explained variance (%)	16.686	11.662	10.969	8.039

The four factors’ initial eigenvalues were 6.331, 4.988, 1.421 and 1.233 respectively, explaining 21.633 %, 16.731 %, 4.895 and 4.097 % of the total variance correspondingly. The percentage of explained variance by factor 2 was 3.42 times of that explained variance by factor 3, approximately equaling to 3.5. According to the psychometrics theory [45], the GDS-30 could also be regarded as two factors. We re-performed the EFA by principal component analysis without rotation, and extracted the fixed two of factors. As a result, factor 1 contained 20 items (item 2, 3, 4, 6, 8, 10, 11, 12, 13, 14, 16, 17, 18, 20, 22, 23, 24, 25, 26 and 28) representing depression, with factor loadings ranging from 0.668 to 0.367, and factor 2 contained 10 items (item 1, 5, 7, 9, 15, 19, 21, 27, 29 and 30) representing positive mood, with factor loadings ranging from 0.779 to 0.438 as shown in Table 4.

**Table 4:** Factor loadings for EFA without rotation in sample 1

Item	Depression	Positive mood
18. Worry a lot about the past	0.668	
25. Frequently feel like crying	0.640	
6. Bothered by thoughts	0.639	
17. Feel pretty worthless the way	0.625	
10. Often feel helpless	0.623	
11. Often get restless and fidgety	0.622	
4. Often get bored	0.617	
24. Frequently get upset over little things	0.616	
8. Afraid something bad is going to happen	0.602	
22. Situation is hopeless	0.600	
16. Often feel downhearted and blue	0.595	
3. Feel life is empty	0.594	
13. Frequently worry about the future	0.583	
2. Drop many activities and interests	0.520	
12. Prefer to stay at home, rather than going out and doing new things	0.518	
26. Have trouble concentrating	0.481	
28. Prefer to avoid social gatherings	0.460	
20. Hard to get started on new projects	0.442	
23. Most people are better off	0.421	
14. Have more problems with memory than most	0.367	
21. Feel full of energy		0.779
9. Feel happy most of the time		0.766
27. Enjoy getting up in the morning		0.765
7. In good spirits most of the time		0.763
19. Find life very exciting		0.712
5. Hopeful about the future		0.708
29. Easy to make decisions		0.689
15. Wonderful to be alive now		0.684
30. Mind as clear as it used to be		0.652
1. Basically satisfied with life		0.438
Explained variance (%)	21.633	16.731

### Confirmatory factor analysis

The CFA was conducted for two-factor models in sample 2, the participants with screened MCI, and the whole sample respectively. The results were reported in Tables 5 and 6. All of the models in these three populations had good fits, and the standardized factor correlation were 0.021, 0.120, and 0.030, respectively.

**Table 5:** Goodness-of-fit indices of confirmatory factor analysis

Models	WLSMV χ^2^	df	*P*	CFI	TLI	RMSEA (90 % CI)
Sample 2	1368.893	404	< 0.0001	0.959	0.956	0.036 (0.034, 0.038)
Participants with screened MCI	2036.067	404	< 0.0001	0.915	0.909	0.045 (0.043, 0.047)
Whole sample	7680.708	404	< 0.0001	0.957	0.954	0.038 (0.037, 0.039)

*WLSMV* robust weighted least squares with mean and variance adjustment, *CFI* comparative fit index, *TLI* Tucker-Lewis index, *RMSEA* root mean square error of approximation

**Table 6:** Standardized factor loadings of the two-factor models in sample 2, screened MCI patients, and the whole sample

Item	Sample 2		Participants with screened MCI		Whole sample	
	Depression	Positive mood	Depression	Positive mood	Depression	Positive mood
2	0.727		0.640		0.674	
3	0.837		0.789		0.802	
4	0.832		0.734		0.820	
6	0.829		0.794		0.836	
8	0.814		0.807		0.815	
10	0.840		0.829		0.846	
11	0.832		0.830		0.826	
12	0.726		0.754		0.738	
13	0.790		0.822		0.804	
14	0.588		0.529		0.556	
16	0.827		0.853		0.848	
17	0.850		0.810		0.822	
18	0.859		0.816		0.847	
20	0.643		0.651		0.664	
22	0.805		0.760		0.792	
23	0.641		0.647		0.664	
24	0.860		0.841		0.849	
25	0.874		0.864		0.868	
26	0.661		0.673		0.682	
28	0.684		0.683		0.661	
1		0.658		0.718		0.687
5		0.810		0.759		0.811
7		0.884		0.840		0.866
9		0.894		0.874		0.888
15		0.764		0.717		0.792
19		0.827		0.817		0.825
21		0.900		0.882		0.885
27		0.863		0.819		0.828
29		0.820		0.755		0.783
30		0.817		0.748		0.797
Factor correlations	0.021		0.120		0.030	

## Discussion

The present study verifies the reliability and construct validity of the GDS-30 for Chinese elderly with screened normal cognition, and evaluates the appropriateness of the GDS-30 used as a screening instrument for depressive symptoms among participants with screened MCI, and in the large-scale community-dwelling general elderly in China.

The results show that the internal consistency of the GDS-30 is satisfactory in the elderly with normal cognition (KR20 = 0.834), in the participants with screened MCI (KR20 = 0.821), and in the large-scale community-dwelling general elderly (KR20 = 0.840). These findings are in line with previous studies using distinct languages of GDS-30 [20–22, 24, 25, 30, 33], and indicate that the reliability of self-reported depressive symptoms by GDS-30 does not change as a function of MCI.

The acceptable explained variance is at least 60 % in the field of social sciences. The total explained variance in our results is relatively small (47.356 %), which cannot be attributed to our sample’s characteristics. In line with our results, other factor analysis studies in this field also found that the explained variance was small, ranging from 38.3 to 63.36 %.

EFA-related results reveal that GDS-30 can be interpreted in terms of four factors: positive mood, dysphoria, worry and social withdrawal-cognitive impairment. We briefly summarize the previous studies on the factor structure of the GDS-30, as shown in Table 7. From the component names, we can see that each factor’s interpretation is very contrived. Firstly, the factors with the same meaning may have different names. For instance, factor 4 ‘mental impairment’ in Adams’s study and factor 5 ‘decreased concentration’ in Parmelee’s study seem to be similar to the factor ‘cognitive impairment’ in other studies in meaning. They are just literal differences. Secondly, the same items of GDS-30 may be interpreted miscellaneously in different studies. Take items 9 ‘Do you feel happy most of the time?’ for example, it is pertained to the factor ‘dysphoria’ in Parmelee’s [33], Adams’s [34] and Hall’s [37] studies, to the factor ‘positive mood and optimism’ in Sheikh’s study [46], to the factor ‘depressed mood’ in Salamero’s study [28], to the factor ‘life dissatisfaction’ in Abraham’s study [35], to the factor ‘hopelessness’ in Bentz’s study [32], to the factor ‘apathy’ in Havins’s study [31], to the factor ‘positive mood’ in Huang’s study [30], and to the factor ‘depression’ in He’s study [27]. It is ambiguous to interpret. Thirdly, the factors of dysphoria, social withdrawal, apathy, cognitive impairment, positive mood are most commonly reported across different studies, which should be considered as different factors of GDS-30 independently. However, they are mixed in some studies due to the EFA, such as factor ‘withdrawal/apathy’, ‘social withdrawal/decreased motivation’, ‘withdrawal-apathy and (lack of) vigor’, etc. Finally, many items are incongruous with their corresponding factors. For example, factor 3, containing items 20, 11, 29 and 28, is defined as apathy in Huang’s study. However, item 28 ‘do you prefer to avoid social gatherings?’ was obviously pertained to the factor of social withdrawal. And factor 7 comprises items 26, 14 and 17. Two of these correspond to cognitive impairment, but the presence of item 17 (do you feel pretty worthless the way you are now?) is clearly inappropriate here.

**Table 7:** Summary of previous studies on factor structure of the geriatric depression scale with 30 items

First author	Analysis	Sample size (n) and description	Components, n	Component names and items	Explained variance, %
Parmele, 1989 [33]	EFA	417, nursing home and congregate apartment residents	6	(1) Dysphoria, (2) Worry, (3) Withdrawal/Apathy, (4) Vigor, (5) Decreased Concentration, (6)Anxiety	52.3 %
Sheikh, 1991 [45]	EFA	326, community-dwelling elderly, ages 66–72	5	(1) Sad mood and pessimistic outlook, (2) lack of mental and physical energy, (3) positive mood and optimism, (4) agitation or restlessness, (5) social withdrawal	42.9 %
Salamero, 1992 [28]	EFA	234, ages 60–95	9	(1) depressed mood, (2) Cognitive impairment, (3) social withdrawal and avoidance, (4)-(9) unnamed	59 %
Abraham, 1994 [35]	EFA, with 26 items	917, nursing home residents, ages 71–97	6	(1) life dissatisfaction, (2) dysphoria, (3) hopelessness/decreased self-attitude, (4) rumination/anxiety, (5) social withdrawal/decreased motivation, (6) decreased cognition	55.1 %
Ertan, 2000^a^ [15]	EFA	276, community-dwelling Turkish elderly and patients with major depression, ages ≥ 60	7; 2	(1) general depressive mood and thought content, (2) life satisfaction, (3) cognitive impairment, (4) energy level, (5) daily outlook, (6) pessimistic view of future, (7) social withdrawal or avoidant behavior; (1) depressive affect and thought content, (2) lack of interest and energy, decrease in performing social and personal activities, and impairment in concentration, memory, and decision-making ability	57.3 %; 36.1 %
Adams, 2001 [34]	EFA	272, Medicare First members of health maintenance organization (HMO), ages ≥ 65	6	(1) Dysphoria 1, (2) Withdrawal-Apathy and (Lack of) Vigor, (3) Anxiety, (4) Mental Impairment, (5) Dysphoria 2, (6) Agitation	50.4 %
Adams, 2004 [29]	CFA, with 26 items	294, independent-living communities, ages 60–98	5	(1) Dysphoric Mood, (2) Withdrawal-Apathy-Lack of Vigor, (3) Anxiety or worry, (4) Cognitive impairment, (5) Hopelessness	-
Chau, 2006 [22]	CFA	253, patients following stroke, ages 72.65 ± 9.36 for female, 70.52 ± 11.04 for male	1	-	-
Bentz, 2008 [32]	EFA	158, geriatric inpatient unit patients, ages 65–101	5	(1) hopelessness, (2) social isolation, (3) negative affect, (4) irritability, (5) worry	44.4 %
He, 2008 [24]	EFA, with 10 items	412, community-dwelling elderly in Hunan province, ages 60–92	6	unnamed	54.670 %
Pocinho, 2009 [17]	EFA, with 27 items	660, Portuguese, ages 65–74	3	(1) Well-being/ill-being, (2) sad mood, (3) mental and physical problems	43.4 %
Hall, 2010 [37]	EFA	173, community-dwelling patients with cognitive impairment (clinical sample), ages ≥ 58	4	(1) Dysphoria, (2) Meaninglessness, (3) Apathy, (4) Cognitive impairment	38.3 %
Havins, 2012 [31]	EFA	569, probable AD patients, ages 39–93	4	(1) Apathy, (2) Dysphoria, (3) Social withdrawal, (4) Cognitive impairment	61.54 %
Liu, 2013 [25]	EFA, with 18 items	397, community-dwelling residents in Beijing, ages 60–97	8	unnamed	53.789 %
Zhang, 2016 [26]	EFA, CFA	420, community-dwelling residents in Sichuan province, ages ≥ 60	6	unnamed	46.848 %
Huang, 2017 [30]	EFA	240, very mild to moderate dementia patients	7	(1) Dysphoria, (2) Positive mood, (3) Apathy, (4) Hopelessness, (5) Social withdrawal, (6) Decreased concentration, (7) Cognitive impairment	59.31 %
He, 2018 [27]	EFA, CFA	1553, community-dwelling residents from three provinces in China, ages 60–99	3	(1) Depression, (2) Apathy, (3) Vigor	63.36 %

*EFA* exploratory factor analysis, *CFA* confirmatory factor analysis

^a^Ertan did the EFA twice, extracting different number of factors

A meta-analysis, which includes 26 published studies using EFA with 14,669 participants who speak 10 languages, provides strong evidence of language differences in the GDS factor structure [18]. Nevertheless, even if the scale with the same language is used, the factor structures are varied tremendously among the Chinese elderly.

In our study, it is reasonable to interpret factor 1 as positive mood, for the 10 items of factor 1 are all positively described. The remaining 20 items, which constitute the other 3 factors, are all negatively described and reflect the depressive mood and thought content. In terms of the difficulty in naming the 3 factors and the high correlation coefficients between the 3 factors, we reconducted the EFA to determine whether two main factors could explain the GDS-30 by extracting the fixed 2 factors in the elderly with normal cognition. As we expect, all of the 30 items clearly loaded 2 factors, namely depression and positive mood, explaining 38.364 % of the total variance. This two-factor structure is much easier to interpret, and the correlation coefficient of these 2 factors is satisfactorily low. The two-factor structure of the GDS-30 can be useful for a better understanding of the epidemiological characteristics of depression in the Chinese elderly. The same findings were found among Turks in Ertan’s study [15].

Most previous studies validating the reliability and validity conducted in Chinese elderly ignored the potential cognitive characteristics [20–27]. Huang’s study explores the factor structure of GDS-30 in patients with very mild to moderate dementia [30]. Our study provides important materials for the appropriateness of depression screening among large-scale community-dwelling Chinese elderly with or without MCI. From the results of CFA results, the two-factor model fit well in the elderly with normal cognition, the participants with screened MCI, and the general community-dwelling elderly. Among these three populations, all of the factor loadings were high (> 0.52), and the goodness-of-fit indices were satisfactory. These indicate that the construct validity of GDS-30 does not change as the cognitive function. The GDS-30 can be recommended as a screening instrument for depression regardless of the presence of MCI.

The first advantage of our study is the utilization of a large community-dwelling general Chinese elderly. The second advantage is that we perform EFA firstly to extract the factors, and then conduct the CFA to confirm the factor structure. Thirdly, we compare the appropriateness of the GDS-30 in elderly with normal cognition, the participants with screened MCI, and the community-dwelling general elderly. Finally, we use MoCA replacing Mini-Mental State Examination (MMSE) to assess participants’ cognitive function. Because MoCA is specially designed for screening MCI, it is more accurate than MMSE when distinguishing the cognitively normal elderly and MCI elderly. Limitation is also of note. The purpose of this large project CCSNSD is to establish the model for assessing the risk of neurological diseases and provide a scientific basis for the development of precise prevention and intervention strategies at the community and individual levels. Since it is not specifically designed to verify the reliability and validity of the GDS-30, we can only analyze the internal consistency and construct validity. The sensitivity and the specificity cannot be analyzed.

## Conclusions

In light of the above, it seems reasonable to conclude that the GDS-30 has good reliability and validity in the Chinese elderly. The two-factor structure is more informative and easier to interpret than the four-factor structure. The GDS-30 can be appropriately applied to screen depressive symptoms in community-dwelling general Chinese elderly regardless of the presence of MCI.

## Data Availability

The datasets used and analyzed during the current study are available from Zhihong Wang, one of the co-authors of this manuscript, on reasonable request.

## Abbreviations

GDS: Geriatric Depression Scale
GDS-30: Geriatric Depression Scale with 30 items
YLDs: Years Lived with Disabilities
BDI: Beck Depression Inventory
HAM-D: Hamilton Depression scale
CES-D: Center for Epidemiological Studies Depression scale
PHQ-9: Patient Health Questionnaire-9
CSDD: Cornell Scale for Depression in Dementia
SDS: Zung Self-rating Depression Scale
EFA: Exploratory Factor Analysis
CFA: Confirmatory Factor Analysis
CCSNSD: Community-based Cohort Study on Nervous System Diseases
MoCA: Montreal Cognitive Assessment
MMSE: Mini-Mental State Examination
WLSMV: Weighted Least Squares with Mean and Variance adjustment
CFI: Comparative fit index
TLI: Tucker-Lewis index
RMSEA: Root mean square error of approximation
SRMR: Standardized Root Mean square Residual
